# Biofilms formed by the archaeon *Haloferax volcanii* exhibit cellular differentiation and social motility, and facilitate horizontal gene transfer

**DOI:** 10.1186/s12915-014-0065-5

**Published:** 2014-08-14

**Authors:** Scott Chimileski, Michael J Franklin, R Thane Papke

**Affiliations:** Department of Molecular and Cell Biology, University of Connecticut, Storrs, Connecticut USA; Department of Microbiology and Center for Biofilm Engineering, Montana State University, Bozeman, MT USA

**Keywords:** biofilm, *Haloferax volcanii*, microbial development, archaeal genetics, archaeal biofilm, horizontal gene transfer, amyloid, collective behavior, swarming, haloarchaea

## Abstract

**Background:**

Archaea share a similar microbial lifestyle with bacteria, and not surprisingly then, also exist within matrix-enclosed communities known as biofilms. Advances in biofilm biology have been made over decades for model bacterial species, and include characterizations of social behaviors and cellular differentiation during biofilm development. Like bacteria, archaea impact ecological and biogeochemical systems. However, the biology of archaeal biofilms is only now being explored. Here, we investigated the development, composition and dynamics of biofilms formed by the haloarchaeon *Haloferax volcanii* DS2.

**Results:**

Biofilms were cultured in static liquid and visualized with fluorescent cell membrane dyes and by engineering cells to express green fluorescent protein (GFP). Analysis by confocal scanning laser microscopy showed that *H. volcanii* cells formed microcolonies within 24 h, which developed into larger clusters by 48 h and matured into flake-like towers often greater than 100 μm in height after 7 days. To visualize the extracellular matrix, biofilms formed by GFP-expressing cells were stained with concanavalin A, DAPI, Congo red and thioflavin T. Stains colocalized with larger cellular structures and indicated that the extracellular matrix may contain a combination of polysaccharides, extracellular DNA and amyloid protein. Following a switch to biofilm growth conditions, a sub-population of cells differentiated into chains of long rods sometimes exceeding 25 μm in length, compared to their planktonic disk-shaped morphology. Time-lapse photography of static liquid biofilms also revealed wave-like social motility. Finally, we quantified gene exchange between biofilm cells, and found that it was equivalent to the mating frequency of a classic filter-based experimental method.

**Conclusions:**

The developmental processes, functional properties and dynamics of *H. volcanii* biofilms provide insight on how haloarchaeal species might persist, interact and exchange DNA in natural communities. *H. volcanii* demonstrates some biofilm phenotypes similar to bacterial biofilms, but also has interesting phenotypes that may be unique to this organism or to this class of organisms, including changes in cellular morphology and an unusual form of social motility. Because *H. volcanii* has one of the most advanced genetic systems for any archaeon, the phenotypes reported here may promote the study of genetic and developmental processes in archaeal biofilms.

**Electronic supplementary material:**

The online version of this article (doi:10.1186/s12915-014-0065-5) contains supplementary material, which is available to authorized users.

## Background

The perception of bacteria and archaea as autonomous single-celled organisms is steadily shifting as the capacity to live within structured communities known as biofilms is discovered in species spanning broad taxonomic groups [1–3]. Biofilms are composed of many microbial cells, often of multiple species, held within an extracellular matrix (ECM) [4]. Because biofilms bring cells together in close physical proximity, the process of biofilm formation is coupled with additional social systems and mechanisms fundamental to microbiology, including quorum sensing (and other forms of cell-to-cell communication), horizontal gene transfer (HGT) and the secretion of enzymes that degrade complex material, causing the ECM to act like a kind of shared external digestive system [3,5]. The end result is a community lifestyle whereby cells may benefit from the advantages of enhanced protection from natural eukaryotic predators [6], other antagonistic microbial species, chemicals, antibiotics or immune responses [5,7,8]. Biofilms may aid in nutrient acquisition and storage [5], facilitate accelerated rates of recombination [9,10] and allow for coordinated multicellular behaviors [11–15]. Direct analyses of aquatic ecosystems have led to the realization that a majority of microbial species in nature exist in biofilms [2,16–20].

General features of bacterial biofilms have been revealed through the study of several key model species, including *Pseudomonas aeruginosa*, *Staphylococcus aureus* and *Bacillus subtilis.* Chemical signals and other external factors often regulate the biofilm lifecycle in bacteria, a sequential process typified by initial attachment of planktonic cells, microcolony formation, maturation into larger structures innervated by aqueous pores or channels, and eventual breakdown or dispersal [5,21,22]. Rather than being simple aggregates of many cells, biofilms contain microenvironments with physical and chemical gradients that establish spatial and temporal genetic patterns sometimes leading to differentiation into multiple cell types [23–26]. Many genes involved in the production and maintenance of matrix materials or extracellular polymeric substances (EPSs) have also been identified [5,27–29]. The principal components of bacterial matrices are polysaccharides, extracellular DNA (eDNA) and amyloid protein [5]. The exact composition, physical and chemical properties, and amounts of these components varies in different species and environmental conditions [4].

While biofilm formation is best characterized for bacterial species [30,31], it has been demonstrated in a number of archaeal groups within the phyla Crenarchaeota and Euryarchaeota, such as *Sulfolobus* spp. [32–34], methanogens [30], acidophilic thermoplasmatales [35], the cold-living SM1 strain found in sulfuric springs [36,37] and halophilic archaea [38]. A recent survey of biofilm formation in haloarchaea (i.e., members of the class Halobacteria) conducted by Fröls and coworkers showed that a majority of tested strains were able to adhere to glass and form biofilms [38]. Species were categorized according to adhesion strength and overall biofilm structure. *Haloferax volcanii* fell within the highest adherence group, and formed large surface associated aggregates, relative to other species, including *Halobacterium salinarum*, which formed smaller micro- and macrocolonies in carpet-like layers [38].

We conducted a physiological analysis of biofilm formation by *H. volcanii* DS2 due to several advantages of using this species as a model for archaeal biofilm formation. The wild-type *H. volcanii* DS2 strain was cultivated from sediment from the Dead Sea in 1975 [39]: it is a relatively fast-growing non-fastidious mesophile, requiring no special equipment to grow in the laboratory [40,41] and was the first archaeon to be artificially transformed [42]. *H. volcanii* DS2 has an available genome sequence [43] and an expanding genetic and proteomic toolbox [42,44–49]. Haloarchaea also undergo promiscuous gene transfer in the environment [50–52] and are excellent species for studying evolutionary processes due to island-like distribution [53–55]. We hypothesize that a cell-to-cell contact-dependent gene transfer mechanism in *H. volcanii* [56–58] may be active when cells are contained within biofilm communities. The available genetic system allowed us to engineer a *H. volcanii* strain expressing GFP for three-dimensional biofilm imaging by confocal laser scanning microscopy (CLSM). Here we characterize key aspects of *H. volcanii* biofilm structural development, composition, dynamics and recombination frequency*.*

## Results

### *Haloferax volcanii* cells develop into structured colony biofilms and static liquid biofilms

**Figure 1:**
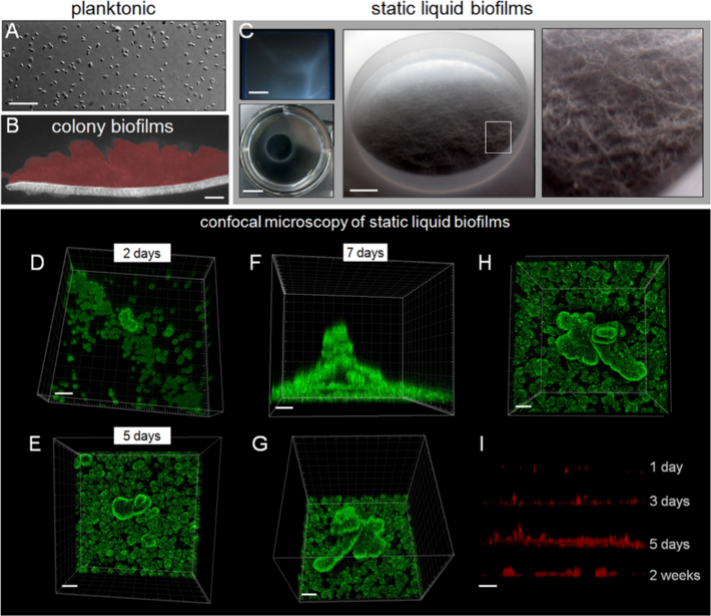
**Growth and development of** ***Haloferax volcanii*** **static liquid biofilms. (A)** Cells within typical shaken culture of *H. volcanii* DS2 under transmitted light. Scale bar equals 10 μm. **(B)** Cross section of cryo-processed *H. volcanii* colony biofilm grown on CA medium for 5 days and stained with CellMask Orange (CMO). Scale bar equals 30 μm. **(C)** Photographs of SL-biofilms routinely grown and analyzed within chamber slides (top left; scale bar equals 2 mm) and on glass coupons within six-well plates (bottom left; scale bar equals 1 cm). A macroscopic photograph of biofilm growth on a 12.7-mm glass coupon is shown in the center (scale bar equals 2 mm) with an area magnified on the right (shown in white box). **(D-H)** CLSM of biofilms grown on glass coupons (within a six-well plate in Hv-Ca medium; scale bars equal 30 μm). Biofilms stained with FM 1-43 were imaged directly in bulk Hv-starve medium using a 63× water-immersion objective after 2 days **(D)**, 5 days **(E)** and 7 days **(F-H)**. **(I)** Biofilm development shown through orthogonal views of SL-biofilms on glass coupons stained with CMO. CLSM, confocal laser scanning microscopy; CMO, CellMask Orange; SL-biofilm, static liquid biofilm.

Planktonic *H. volcanii* DS2 cells grown in shaking culture (Figure 1A) readily formed biofilms in typical rich media types Hv-YPC and Hv-Ca within several experimental systems that provided a solid plastic or glass substratum. Colony biofilms [7] developed on the surface of polycarbonate filters placed on solid media (Figure 1B) and were cryo-processed and cross-sectioned, exposing a surface structure containing crevices bounded by globular structures (Figure 1B). The greatest level of structural complexity was observed when biofilms were grown in static liquid (SL-biofilms; Figure 1C). Cultivating SL-biofilms within chamber slides and on the surface of borosilicate glass coupons placed in six-well plates permitted direct staining and optimized imaging of delicate biofilm structure that was visible macroscopically (Figure 1C).

We began by examining the development of wild-type *H. volcanii* DS2 biofilms in static liquid using CLSM and the cellular membrane dyes FM 1-43 and CellMask Orange (CMO; Figure 1D–I, Additional file 1: Table S1). Circular microcolonies formed within 48 h (Figure 1D), which led to well-defined clusters/aggregates after 5 days (Figure 1E). SL-biofilms reached maturation after 7 days of incubation at 42°C having developed into multi-layer towers with flake-like morphology (Figure 1F,G,H). Large towers were surrounded by a dense layer of smaller clusters or microcolonies and were separated by areas with little or no cell density (Figure 1E–H). Overall structural integrity was maintained as large clusters surrounded by smaller microcolonies for several weeks, although a comparison of orthogonal views of CMO-stained SL-biofilms showed that after 2 weeks the height of most structures was diminished and less of the total surface area was covered with microcolonies (Figure 1I, 2 weeks). Large clusters/towers, like the example shown in Figure 1F,G,H, varied in height and width, with a maximum measured height of 148 μm.

### Microcolonies within biofilms formed by a GFP-expressing *Haloferax volcanii* strain bind stains targeting polysaccharide, DNA and amyloid protein

**Figure 2:**
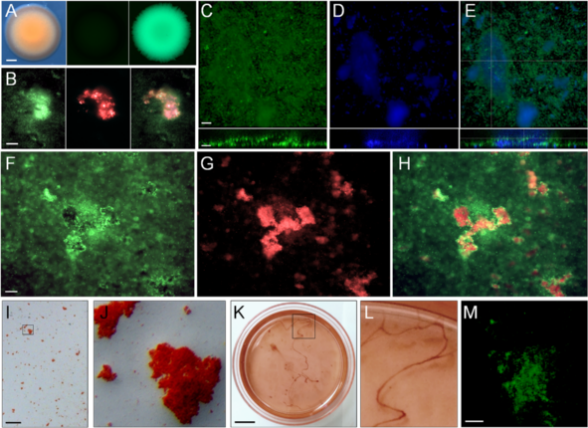
**Visualizing the extracellular matrix of** ***Haloferax volcanii*** **biofilms. (A)** Development of a GFP-expressing strain for use in biofilm visualization: colonies formed by the parental strain *H. volcanii* H1206 under transmitted light (left), under blue excitation (center), and by the strain *H. volcanii* H1206(pJAM1020) under blue excitation (right). Scale bar equals 250 μm. **(B)** Seven-day *H. volcanii* H1206(pJAM1020) SL-biofilm stained with concanavalin A-Texas Red collected with blue excitation (left), green excitation (center), and shown as an overlay (right). Scale bar equals 20 μm. **(C,D,E)** Top-down projections of a Z-stack for a *H. volcanii* H1206(pJAM1020) SL-biofilm stained with DAPI. **(C)** GFP signal under blue excitation. **(D)** DAPI-stained material with violet excitation. **(E)** Overlay of **(C)** and **(D)**. Orthogonal views from CLSM analysis are shown below each panel and the plane of the orthoslice is shown in **(E)**. Scale bars equal 20 μm. **(F,G,H)** Seven-day *H. volcanii* H1206(pJAM1020) SL-biofilm stained with Congo red with blue excitation **(F)**, and green excitation for CR fluorescence **(G)**, with an overlay of **(F)** and **(G)** shown in **(H)**. Scale bar equals 20 μm. **(I)** Bright-field view of 7-day CR-stained SL-biofilm within a chamber slide. Scale bar equals 1 mm. Area outlined by black box is shown in **(J)**. **(K,L)** Ten-day SL-biofilm within Petri dish grown at 25°C in medium containing CR with CR-stained string or web-like structures magnified in **(L)**. Scale bar equals 1 cm. **(M)** Seven-day H1206 SL-biofilms grown in Hv-YPC medium stained with thioflavin T under blue excitation (top down 3D projection of a Z-stack; scale bar equals 20 μm). CLSM, confocal laser scanning microscopy; CR, Congo red; DAPI, 4′,6-diamidino-2-phenylindole; GFP, green fluorescent protein; SL-biofilm, static liquid biofilm.

Several *H. volcanii* strains were engineered to express GFP to study ECM composition with the goal of reinforcing and expanding staining experiments conducted by Fröls and coworkers [38]. Confirmation of GFP expression was first accessed within colonies formed by two GFP-expression strains produced in this study. Colonies formed by the parental *H. volcanii* H1206 strain (Figure 2A; left panel) did not autofluoresce with blue excitation (Figure 2A, center panel), while those formed by the *H. volcanii* H1206(pJAM1020) strain based on the previously developed plasmid pJAM1020 (see Additional file 1: Table S2) [59] showed uniform and high levels of GFP fluorescence (Figure 2A; right panel). An additional GFP-expressing strain *H. volcanii* H1206(pSC409GFP), containing the same red-shifted *gfp* gene as in pJAM1020 but cloned into the plasmid pTA409, produced colonies with GFP signals that differed in both intensity and spatial distribution, resulting in an assortment of patterns of GFP signal within developed colonies (Additional file 1: Figure S1). The H1206(pJAM1020) strain was therefore selected for biofilm compositional studies to ensure stable GFP expression throughout the cellular population.

Cellular clusters visible in GFP-expressing biofilms were colocalized with the signal from a Texas Red conjugate of the lectin concanavalin A (ConA; Figure 2B) and with the DNA binding dye 4′,6-diamidino-2-phenylindole (DAPI; Figure 2C,D,E). ConA is known to bind a-manopyranosyl and a-glucopyranosyl residues within glyconjugates of haloarchaeal biofilms [38]. DAPI was selected as an extracellular DNA stain for our CLSM study because it is known to stain eDNA preferentially in haloarchaeal biofilms [38]. Fröls and coworkers [38] used three nucleic acid dyes to distinguish between extracellular and intracellular DNA in biofilms formed by *H. volcanii* and additional haloarchaeal strains, and showed that: (a) only acridine orange stained individual live cells, appearing similar our use of the cell permeable nucleic acid dye SYTO 9 (Additional file 1: Figure S2), (b) the signal from DAPI appeared nebulous and granular and was colocalized with microcolonies, (c) simultaneous staining with 7-hydroxy-9H-1-3-dichloro-9,9-dimenthylacridin-2-one (DDAO), a nucleic acid dye considered completely impermeable to cells, led to an essentially identical pattern of fluorescence signals, and (d) very few non-viable cells are present within *H. volcanii* biofilms (even after 30 days of incubation), suggesting that the observed DAPI signal was not from dead cells. Our CLSM analysis also showed colocalization of DAPI-stained material with microcolonies and a lack of signal from individual cells. Further three-dimensional reconstruction of DAPI-stained GFP-expressing biofilms revealed that DNA was concentrated in the basal layer of the biofilm and dispersed as plume-like structures at the top of larger towers (Figure 2C,D,E; lower panels).

Larger structures observed in mature biofilms with transmitted light and through GFP fluorescence were also colocalized with the signal from Congo red (CR) and thioflavin T (ThT). These stains are routinely used as characteristic tests for the presence of a wide variety of amyloid proteins, including for diagnosis and study of disease-causing plaques formed by amyloidosis [60–63], and in many investigations of microbial adhesion and biofilm formation [27,64–71].

Congo red fluorescence (CRF) was used as it has been proposed as the most sensitive and reliable method for amyloid detection when staining with CR [60] and has been applied to biofilm compositional studies [72]. CRF was sharply defined and granular in appearance and was colocalized with the large biofilm cellular clusters and towers shown in Figure 2F,G,H. Overlays where single GFP-producing cells are visible and images of CRF at 600× magnification (Additional file 1: Figure S3) indicated that CR did not stain individual cells. CR-stained biofilm aggregates were also orange-red under transmitted light and were visible macroscopically (Figure 2I–L). Further, green fluorescence signal was detected within ThT-stained mature biofilms formed by a non-GFP strain with blue excitation (Figure 2M; no signal detected in control without ThT staining).

#### *Haloferax volcanii* undergoes morphological differentiation during biofilm growth

**Figure 3:**
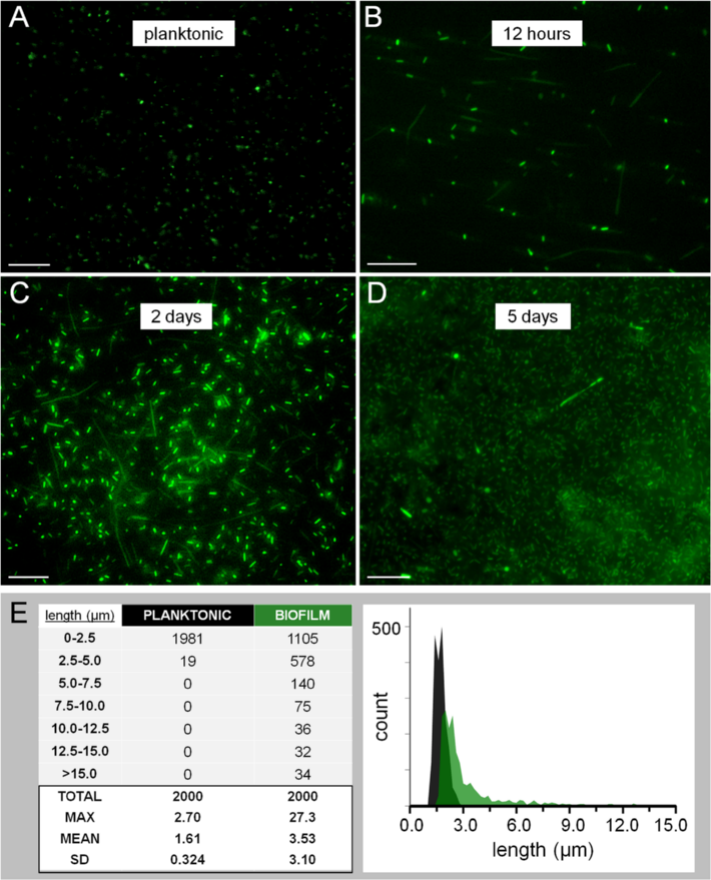
**Cellular morphology within developing** ***Haloferax volcanii*** **H1206(pJAM1020) static liquid biofilms. (A)** Planktonic *H. volcanii* H1206(pJAM1020) cells from exponential phase shaking Hv-YPC culture. **(B,C,D)** Cells within a developing H1206(pJAM1020) SL-biofilm grown in a chamber slide (in Hv-YPC medium) after 12 h **(B)**, 2 days **(C)** and 5 days **(D)**. **(E)** Table listing the number of cells binning into 2.5-μm categories and summarized statistics for 2,000 cells measured for planktonic and biofilm cell populations (left) and histogram showing distribution of length at 0.2-μm binning intervals for planktonic (black) and biofilm (green) cells (right). Cell length was measured with Fiji particle analysis using images collected from three independent exponential phase shaking cultures and three 12 h biofilms grown in chamber slides. Mean length of populations was statistically different with *P* < 0.0001. Scale bars equal 20 μm. SD, standard deviation; SL-biofilm, static liquid biofilm.

The implementation of GFP for biofilm visualization led to the unexpected observation of increased variability in length of cellular structures within biofilms compared to planktonic cells (Figure 3). *H. volcanii* DS2 cells are known to be pleomorphic, appearing spherical, disk-like or as short rods 1 to 3 × 2 to 3 μm in size in liquid culture [39] (Figures 1A and 3A). However, during examination of GFP-expressing *H. volcanii* H1206(pJAM1020) biofilm cells, we observed large cellular structures composed of long rods in chains sometimes approaching 30 μm in length (Figure 3B,C,D). These structures were attached to the surface and were found within developing H1206(pJAM1020) biofilms at all observed time points (Figure 3B,C,D; Additional file 2: Movie 1), in several independently transformed H1206(pJAM1020) strains, as well as in biofilms formed by *H. volcanii* strains that do not have the GFP expression plasmid (H53 and H98; see Additional file 1: Figure S2).

Further experimentation verified the relationship between the chained long rod morphotype and biofilm formation in the H1206(pJAM1020) strain. A culture of planktonic cells with an average length of 1.6 μm underwent a morphological shift during the first 12 h of biofilm formation in replicate chamber slide wells (Figure 3E). The difference in length between populations of 2,000 planktonic and 2,000 biofilm cells was statistically significant (*P* < 0.0001 in unpaired *t* test). Cellular structures within biofilms were on average greater than twice as long, were more variable in size (with a standard deviation in length of 3.1 μm) and reached a maximum length ten times greater than that measured within the planktonic culture from which the biofilm was derived (Figure 3E).

#### *Haloferax volcanii* exhibits social motility following disruption of static liquid biofilms

**Figure 4:**
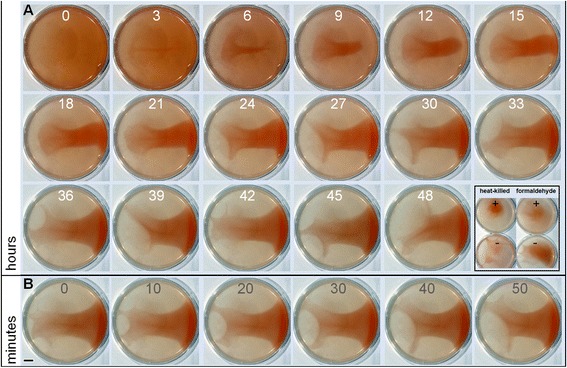
**Time-lapse macroscopic photography of static liquid biofilm reformation.** An established 7-day SL-biofilm grown in a plastic Petri dish in rich medium (Hv-YPC) was mechanically homogenized and left to incubate at 42°C while photographs were taken at regular time intervals. **(A)** Biofilm reformation over a 2-day period, with images shown at 3 h intervals. Inlay: Cellular vitality controls shown as still images from time-lapse series (see Additional files 6 and 7: Movies 5 and 6) of a SL-biofilm treated with heat (left) and with 4% formaldehyde (right). **(B)** SL-biofilm imaged at 10-min intervals over a 50-min period (a series from between 45 h and 48 h above). Scale bar equals 1 cm. SL-biofilm, static liquid biofilm.

An investigation of biofilm dynamics in static liquid began by disrupting mature 7-day biofilms through mechanical homogenization followed by time-lapse photography over a reformation period. To our surprise, this led to the discovery of rapid cellular re-aggregation and sustained coordinated social motility (Figure 4; see Additional files 3, 4, 5, 6 and 7: Movies 2–6). Structured cellular aggregates were visible 3 h following homogenization, after which cell density was incrementally concentrated within a central developing SL-biofilm, with the surrounding medium becoming visibly transparent after 48 h (Figure 4A). Samples were collected from the homogenized biofilm at time zero, and from the central biofilm and surrounding medium at 48 h. The homogenized biofilm was composed of single cells and cellular aggregates; after 48 h, the surrounding medium contained few planktonic cells and the SL-biofilm contained a high density of cells in large aggregates. Images collected during reformation at intervals of 3 h (Figure 4A), 10 min (Figure 4B) and 1 min (see Additional files 3 and 4: Movies 2 and 3) captured the coordination of large filaments of cells into dynamic web-like branches. Rippling or wave-like streaming was observed during extension and retraction of these structures, particularly evident at low frame rates (e.g., see Additional file 4: Movie 3).

Social motility was also triggered by physical agitation or partial disruption of previously undisturbed developing SL-biofilms (see Additional files 5, 6 and 7: Movies 4, 5 and 6). Ring-shaped SL-biofilms, which developed 3 days after an exponential phase culture was left to incubate in replicate Petri dishes under static conditions, formed extensions and migrated towards the perimeter of the dish through a network of apparent cellular streams for over 1.5 h after the culture vessel was gently moved (see Additional file 5: Movie 4). Additional SL-biofilms from the same replicate set retained this sensitivity through day 5 of incubation. As SL-biofilms became denser and reached a stationary phase of growth after 10 days of incubation, identical applications of physical agitation or partial disruption no longer induced social motility. Coordinated motility as shown in Figure 4 and in Movies 2 to 6 (Additional files 3, 4, 5, 6 and 7) has been observed in many independent experiments and is absent in identically prepared heat-treated SL-biofilms (Figure 4A; Additional file 6: Movie 5) and SL-biofilms chemically fixed with formaldehyde (Figure 4A; Additional file 7: Movie 6).

### Genetic transfer occurs at high frequency within *Haloferax volcanii* biofilms

A screen for gene transfer within *H. volcanii* biofilms through the known mating mechanism was conducted by cultivating biofilms composed of two strains, each with a unique auxotrophic marker. Gene transfer frequency of the auxotrophic markers was calculated as the number of colonies forming on selective medium (auxotrophs that had regained prototrophy), divided by the number of total viable cells recovered for each culture condition, as described previously [56,57]. A comparison of transfer frequency was conducted for mixed shaking cultures, colony biofilms and SL-biofilms in chamber slides.

**Figure 5:**
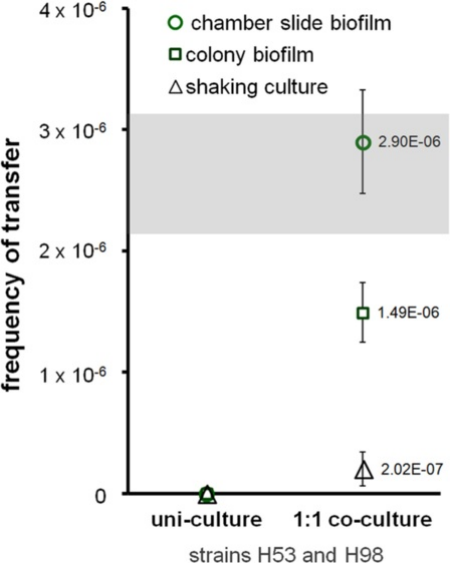
**Recombination within** ***Haloferax volcanii*** **biofilms.** Two double auxotrophic strains (H53, ∆*pyrE2*/∆*trpA*; H98, ∆*pyrE2/*∆*hdrB*) were mixed at equal cell densities and grown together in Hv-YPC + thymidine medium for 7 days under shaking conditions (black triangles), or as colony biofilms (green squares) and chamber slide SL-biofilms (green circles). Cultures and biofilms were then harvested, washed and plated on defined medium with uracil alone, to select for recombinants, which are either H53 cells that have regained tryptophan prototrophy through a transfer event with a H98 cell(s) or H98 cells that have gained thymidine prototrophy from a H53 cell(s). Average frequency of transfer is shown for three replicates per condition. The transfer frequency range reported in the study in which this HGT mechanism was discovered, traditionally conducted using nitrocellulose filters and known as mating, is shown as a grey box [56]. Vertical bars equal one standard deviation. HGT, horizontal gene transfer.

Transfer frequencies indicated that the mating mechanism was active within colony biofilms and SL-biofilms (Figure 5). For chamber slide SL-biofilms, an average transfer frequency of 2.90 × 10^-6^ was measured and is within the range reported in Mevarech and Werczberger’s 1985 study in which mating was first discovered through co-filtering cells on nitrocellulose filters [56]. The transfer frequency for a shaking culture control was low as expected, and mating was not quantifiable in uni-culture biofilms of either H53 or H98 cells (as there is no available source for transfer of the gene required for growth on the selective medium).

Planktonic (shaking) and SL-biofilm cells were visualized through microscopy in parallel prior to plating to determine whether the cellular morphology was consistent with known models for mating, i.e., that it requires cell-to-cell contact and involves the formation of cytosolic bridges and/or cellular fusion events [58]. Cells from a shaken culture were small and disk-shaped while those in chamber slide biofilms had formed a densely packed basal layer of morphologically diverse cellular structures in close physical association (Additional file 1: Figure S2). Natural transformation has not been reported in *H. volcanii*. In fact, an increase in transfer frequency was reported after adding DNase during mating experiments on filters, indicating that gene transfer in *H. volcanii* biofilms does not occur through transformation [56].

## Discussion

### Structural development of *Haloferax volcanii* biofilms and biopolymers of the extracellular matrix

The present study and that of Fröls and coworkers [38] demonstrate a propensity of haloarchaeal species to form biofilms and indicate this may be a predominant natural mode of growth for this group of organisms. While haloarchaea are considered extremophiles, the ability of any microbial group to form biofilms is consistent with the emerging view that biofilm formation is an adaptation common to most if not all species [16–19]. In this particular case, the benefits associated with a community lifestyle match the environmental conditions that challenge microbial life in hypersaline environments. For example, encapsulation within a hydrated nutrient-dense ECM and a biofilm structure with areas that surround developed microcolonies (Figure 1E-H), which likely act as channels to facilitate waste and nutrient exchange, may help explain the persistence of haloarchaeal populations through prolonged periods of starvation [73]. Overall, the delicate and motile nature of multicellular structures imaged in static liquid (Figure 1C-I and Figure 4) may reflect low levels of natural circulation and water activity within hypersaline environments and a selective advantage associated with movement towards favorable conditions.

Our staining experiments with *H. volcanii* H1206(pJAM1020) allowed for accurate discrimination between cellular structures emitting endogenous GFP signal and ECM and support previous evidence showing that polysaccharide and eDNA are major biofilm components (Figure 2B-E) [38]. Species of the genus *Haloferax* have long been known to produce exopolysaccharide [74] and recent studies of *H. mediterranei* (average nucleotide identity of 86.6% with *H. volcanii* [57]) have identified genes essential for exopolysaccharide synthesis and export [75]. Homologs of genes within an *H. mediterranei* operon shown to be required for exopolysaccharide production [75] are present in the *H. volcanii* genome. Several putative EPS operons have been identified: HVO_2056-HVO_2057 on the main chromosome, and HVO_A0216-A0221 and HVO_A0594-0595 on the mini-chromosome pHV4. While the function(s) of extracellular DNA within *H. volcanii* ECM is not yet clear, in bacterial biofilms, eDNA is known to be involved in attachment to surfaces, microcolony formation, overall structural integrity and even spatial self-organization [13,76–78]. In several crenarchaeal species, eDNA is present within biofilms but is not a structural component [32]. Similar attempts at DNase treatment of eDNA in haloarchaeal biofilms were unsuccessful and may have been complicated by high salt concentrations within the growth medium [38]. Interestingly, ECM is also known to be a mechanism for nutrient storage [5], and we have previously demonstrated that *H. volcanii* is capable of utilizing eDNA as a nutrient [79,80], suggesting that eDNA may contribute towards the growth of cells within the biofilm.

Bacterial appendages involved in attachment and biofilm formation are often composed of amyloid proteins [27,64,81,82]. Previous studies have surveyed the presence of amyloid in natural biofilms using the dyes CR and ThT and found that it is an abundant and widely distributed ECM component [66,67]. Those same studies reported that amyloid production by archaea could not be determined due to low recovery of archaeal cells from sample sites [66]. On the basis of visible staining with CR, CRF and ThT fluorescence, we have identified putative amyloid protein that was colocalized with *H. volcanii* microcolonies (Figure 2F-M). Although it is most commonly used to stain amyloid fibrils, it is important to note that CR may stain other biopolymers such as polysaccharide [83]. However, because it was colocalized with microcolonies and larger biofilm structures (i.e., not individual cells), CR likely stained a major ECM component and not an internally stored polymer. The gene HVO_1403 is homologous to a known amyloidogenic factor in *Sulfolobus solfataricus* [84] and is a potential genetic determinant of amyloid protein within *H. volcanii* biofilms.

#### Social motility of static liquid biofilms formed by *Haloferax volcanii* as a multicellular or collective behavior

Macroscopic time-lapse images collected following disruption of SL-biofilms (Figure 4A) demonstrate a propensity for *H. volcanii* cells to re-aggregate within hours, rather than remaining suspended in liquid culture or evenly precipitating. During and after reformation, multicellular filaments or extensions formed, appearing to self-organize and explore the surface area along the bottom of the culture plate (see Additional files 3, 4, 5, 6 and 7: Movies 2–6). This activity was not seen in identically prepared SL-biofilms that had been heat-treated or treated with formaldehyde (see Additional files 6 and 7: Movies 5 and 6, respectively), indicating it is an active biological process, and suggesting the existence of an archaeal system evocative of the elaborate swarming behavior characterized in the soil bacterium *Myxococcus xanthus* [11,85,86] and in *Pseudomonas aeruginosa* [13]*.* Although swarming is strictly defined as migration across a solid surface [87] and is typically imaged in these species at a solid–air interface, early studies of rippling and wave-formation in *M. xanthus* swarms were also conducted in submerged liquid culture [88,89].

The existence of a system for social motility in *H. volcanii* is supported by the known production of type IV pili-like structures responsible for surface adhesion [90], the prior observation of a hypermotile phenotype [91] and the presence of a complete set of chemotaxis genes (including CheA, CheB, CheC, CheD, CheR and CheW) positioned adjacent and interspersed with archaeal flagella gene clusters (i.e., *flgA1* and *flgA2*) [43,90,91]. A predicted operon including four genes on the main chromosome (HVO_1221 to 1224) is of particular interest as HVO_1222, HVO_1223 and HVO_1224 proteins are homologs of bacterial frizzy aggregation (Frz)/defective in fruiting (Dif) proteins FrzF, FrzE/DifE and FrzG, respectively, factors that are essential determinants of social motility and ECM production in *M. xanthus* [92]. The ecological role of social motility in *H. volcanii* is undetermined at this point. It is plausible that multicellular groups might migrate towards a nutrient influx during known bloom events [93], or that social motility is associated with an additional activity, such as cooperative feeding.

#### Haloferax volcanii *biofilms are hotspots for gene transfer*

While *H. volcanii* is often noted for its unique gene transfer system [56], the mechanism and genes underpinning this phenomenon remain largely uncharacterized [57,94]. Here we report that mating occurs within biofilms at the same frequency as in earlier reports (Figure 5), consistent with the known cell-contact-dependent nature of the mechanism. Mating was discovered and has been routinely observed in the laboratory by several groups by co-precipitating cells or co-filtering them on nitrocellulose filters [56–58,94,95]. These experimental methods for bringing cells together in close proximity can be thought of as proxies for naturally formed biofilms.

We show that *H. volcanii* biofilms contain a biologically formed layer of pleomorphic adherent cells at high density and in close association (Figure 3B,C,D and Additional file 1: Figure S2). The observed spatial arrangement and diversity of cellular structure is consistent with the model of cytoplasmic bridge formation and membrane fusion during mating [95], previous electron micrographs showing elongated cells with intercellular structures [39], and/or the involvement of additional contact-dependent cargo-transfer mechanisms not described in *H. volcanii*, such as lipoprotein transfer [96], vesicular trafficking [97] and cell-to-cell secretion systems [98]. Based on these observations, we suggest biofilms are the microenvironments where mating likely occurs in nature, and propose this mode of growth as an excellent way for studying and visualizing HGT mechanisms in live cells and in real time.

#### Cellular morphogenesis as phenotypic reflection of differentiation into distinct genetic cell types

In *Bacillus subtilis*, a distinct chained-rod morphology is associated with a population of cells known to produce ECM and to remain attached to the surface, while short unchained rods express flagellar components and are motile [99]. Here, we identified a population of surface-attached cellular structures appearing during biofilm formation and present within developing biofilms but never in planktonic culture (Figure 3; see also Additional file 1: Figure S2 and Additional file 2: Movie 1). Long rod structures were confirmed as live cells, as the only source of fluorescent signal in Figure 3B,C,D was endogenous GFP. This morphotype is unlikely to be the result of a spontaneous mutation in the H1206(pJAM1020) strain; it was observed in several independent strains and *Haloferax* species are known to have a low spontaneous mutation rate [100].

Our observations are consistent with reports of “irregular” morphological characteristics (labeled as “involution forms” or “hyphomicrobium-like” structures) when *H. volcanii* DS2 is grown on agar medium, as noted in the original 1975 characterization [39]. Archaeosortase (*artA*) overexpression strains also showed an elongated rod morphology. The ArtA protein has many downstream targets effecting cell shape and surface decoration, indicating that it could be a regulatory component of biofilm formation and/or related systems [94]. Previous observations of irregular cell morphologies can now be understood as being coupled with biofilm formation.

Additional cellular functions may also be associated with a chained-rod or adherent cell phenotype. Larsen and Mullakhanbhai [39] found narrow constrictions with an apparently contiguous S-layer connecting cells together like beads on a string. In light of the later discovery of the mating HGT mechanism and electron micrographs of mating cells, the constrictions and associations between GFP-expressing cells observed in biofilms in this study (Figure 3B,C,D; Additional file 1: Figure S2 and Additional file 2: Movie 1) may be viewed as cytoplasmic bridges, which have been proposed as a prerequisite step towards cellular fusion and chromosomal mixing and/or recombination [56–58,95]. Further genetic analyses will shed light on the possible specialized function(s) (e.g., matrix-production or competence for mating) of this cellular phenotype.

## Conclusions

The archaeon *H. volcanii* forms biofilms in several experimental systems that can be visualized in the laboratory using standard protocols developed for bacterial species. *H. volcanii* cells develop into biofilm structures that exhibit complexity in both cellular morphology and ECM composition. We have reported several key features of *H. volcanii* biofilms: structural development through microcolony formation and maturation phases, visualization of ECM, including the identification of putative amyloid fibrils, an archaeal form of social motility, as well as a chained-long rod morphotype and gene transfer. With the implementation of fluorescent proteins for biofilm visualization and the available genetic system, it is clear that the study of biofilm formation in *H. volcanii* is not only a means for understanding haloarchaeal ecology and physiology, but also acts as an excellent model for the molecular biology of archaeal biofilms and multicellular behaviors. Future studies may focus on the genetic identification of many possible differentiated cell types and the visualization of their positions in time and space using fluorescent protein reporters. The likely many genetic determinants of ECM components, social motility and the enigmatic mating mechanism for gene transfer in *H. volcanii* all remain to be discovered.

## Methods

### Strains and culture media

*H. volcanii* strains (see Additional file 1: Table S2) were provided by Thorsten Allers of the University of Nottingham, UK, and grown in media adapted from previous studies [47]. Rich medium (Hv-YPC) contained 144 g NaCl, 21 g MgSO_4_ × 7H_2_O, 18 g MgCl_2_ × 6H_2_O, 4.2 g KCl, 12 mM Tris-HCl pH 7.5, 3.125 ml 1 M CaCl_2_ solution, 1.0 ml trace element solution, 5.0 g yeast extract, 1.0 g casamino acids, and 1.0 g peptone per liter. Hv-YPC used to culture GFP biofilms for EPS staining also contained 0.5% glucose.

Hv-Ca medium contained 144 g NaCl, 21 g MgSO_4_ × 7H_2_O, 18 g MgCl_2_ × 6H_2_O, 4.2 g KCl, 12 mM Tris-HCl pH 7.5, 3.125 ml 1 M CaCl_2_ solution, 1.0 ml trace element solution, 5.0 g casamino acids, 0.125 ml 6.4 mg/ml thiamine solution, and 0.125 ml 0.8 mg/ml biotin solution per liter.

A basal-salts starvation medium (Hv-starve) contained all components in Hv-YPC other than complex nutrients (i.e., yeast extract, casamino acids and peptone). Solid media contained 18 g of agar per liter. When required, uracil was provided at 50 μg/ml (∆*pyrE2* strains), thymidine and hypoxanthine at 40 μg/ml (∆*hdrB* strains), tryptophan at 50 μg/ml (∆*trpA* strains), and novobiocin at 0.30 μg/ml for antibiotic selection.

### Development of GFP-expressing *Haloferax volcanii* H1206 strain

Two *H. volcanii* uracil auxotrophic strains were developed for constitutive GFP-expression and cell imaging by CLSM and epifluorescence microscopy (see Additional file 1: Table S2). The strain H1206(pJAM1020) was based on selection through novobiocin resistance using a previously developed vector [59], and a second strain H1206(pSC409GFP) was based on the gene *pyrE2* and uracil autotrophy [44,46,47]. Plasmid pSC409GFP was constructed by amplifying the region from pJAM1020 containing red-shifted GFP and P2 promoter for constitutive expression in haloarchaeal species [59], and inserting this construct into the shuttle vector pTA409 [101] (see Additional file 1: Tables S2 and S3). Each plasmid was transformed into the strain H1206 (Δ*pyrE2* Δ*mrr*) using the PEG-based method as previously developed [49,102,103], and transformants were selected on Hv-Ca medium without uracil for H1206(pSC409GFP) or containing novobiocin for H1206(pJAM1020). *H. volcanii* H1206 is identical to H26 other than an additional deletion of the *mrr* gene, which encodes a restriction endonuclease that cleaves methylated foreign DNA, allowing transformation without passage through an *Escherichia coli dam* mutant [46]. Growth kinetics of each GFP strain in liquid Hv-YPC medium were measured by optical density and were unchanged from the parental strain.

### Biofilm growth systems

The media described above were prepared and used to cultivate *H. volcanii* biofilms using the experimental growth methods described below. All biofilms were incubated at 42°C unless otherwise stated.

#### Colony biofilms

Colony biofilms as shown in Figure 1B were prepared on 13-mm polycarbonate filters (0.2-μm pore size, Sigma, St. Louis, MO, Z365041) as previously described [7,23]. Diluted *H. volcanii* culture (OD _600 nm_ = 0.1) was transferred onto the surface of sterile filters previously placed directly on the surface of the solid medium with sterile forceps. After the culture medium penetrated the filter and evaporated, the plates were inverted and incubated. Filters were transferred to fresh plates under sterile conditions every 48 h during the growth period.

#### Static liquid biofilms

SL-biofilms were set up by aliquoting inoculated growth medium into six-well plates and Petri dishes (as in Figures 1C, 2K and 4, and see Additional files 3, 4, 5, 6 and 7: Movies 2–6), or chamber slides (Lab-Tek II, with 0.7 cm^2^ growth area per well; as in the top left panel of Figure 1C and Figure 2I,J). A borosilicate glass coupon (Biosurfaces Technologies Corporation, Bozeman, MT, RD 128-GL) or a piece of coverglass was placed inside the six-well plates (as in Figure 1C) to provide a biofilm growth surface that could be visualized directly through microscopy.

### Biofilm staining and microscopy preparation

The properties and experimental parameters used for all dyes in this study are summarized in Additional file 1: Table S1. The final concentration of each staining solution was optimized based on either the manufacturer’s protocol or a previous study. CR staining was performed using the alkaline CR method [104,105]. Biofilms were stained and prepared for microscopy as described below for each growth method.

#### Colony biofilms

Mature colony biofilms were stained by transferring a staining solution in Hv-starve medium directly on top of the colony biofilm and incubating at room temperature for 60 min. Cross sections of stained colony biofilms (e.g., Figure 1B) were cryo-processed by rapid freezing on dry ice and cross-sectioned using a Leica CM1850 cryostat microtome as described previously [23]. Then, the 5-μm transects were placed onto microscope slides and visualized immediately.

#### Glass surfaces within SL-biofilms

Staining on glass coupons and coverglass was conducted by removing the growth substrate from the growth system/medium, followed by placement in fresh sterile 60-mm Petri dishes. An Hv-starve staining solution was then gently transferred (immediately, before the remaining culture medium on the surface of the glass evaporated) to the surface of the substrate (i.e. glass coupon), completely covering the surface. This volume of staining solution remained on the surface through surface tension during a 60-min staining period at room temperature in the dark. The staining solution was then carefully removed and the surface was washed once with Hv-starve (same volume as used for staining solution). The substrate was then placed in a new Petri dish and fresh Hv-starve medium was added until the surface of the growth surface was submerged by approximately 5 mm.

#### Chamber slides

Biofilms grown in chamber slides were stained by removing conditioned medium from the wells through gentle aspiration, followed by the addition of Hv-starve staining solution (same volume as medium used to grow biofilm, e.g. 500 μl) for 60 min at room temperature in the dark. Staining solution was then removed, the surface was gently washed once with the addition and subsequent removal of another equal volume of Hv-starve, and fresh Hv-starve was added, followed by direct visualization.

### Fluorescence microscopy and imaging

#### Confocal microscopy

An upright Leica Microsystems (Wetzlar, Germany) TCS SP5 laser-scanning confocal microscope was used (for data shown in Figure 1D-I) with a 63× water immersion lens placed directly into the bulk fluid above biofilms grown on glass coupons or coverglass. Images were acquired through the Leica Application Suite Advanced Fluorescence software platform. Three-dimensional reconstructions of focal plane slices were generated and analyzed using IMARIS from Bitplane (Zürich, Switzerland; Figure 1D-H) or Fiji [106]. An Andor spinning disk confocal system with a Nikon Eclipse Ti microscope (data shown in Figure 2C,D,E,M) and Nikon A1R Spectral Confocal Microscope (Additional file 1: Figure S2) were also used to image live biofilms within chamber slides.

#### Two-dimensional microscopy

Images were collected using an upright Nikon Eclipse E800 epifluorescent microscope, also with water immersion lenses placed directly above cultured biofilms. For biofilms grown in chamber slides, they were collected with an inverted Nikon Eclipse TE300 with a SPOT digital microscope camera (shown in Figures 1A,B, 2B,F-H and 3A-D, and Additional file 1: Figures S1,S2,S3 and Additional file 2: Movie 1).

#### Macroscopic imaging

Colonies and SL-biofilms in chamber slides were imaged using a Zeiss Discovery V20 stereo fluorescence dissecting microscope with a Zeiss AxioCam camera (Figures 1C and 2A,I-L and Additional file 1: Figure S1). Photographs of live SL-biofilms in time-lapse studies were taken using a digital single-lens reflex camera and macro lens, LED light source and intervalometer mounted within an incubator (Figure 4 and Additional files 3, 4, 5, 6 and 7: Movies 2–6).

#### Additional imaging details

No autofluorescence signals were detected from *H. volcanii* cells or biofilm structures in all excitation channels used in this study (for excitation wavelengths used for each dye, see Additional file 1: Table S1). Images collected with black and white digital cameras were pseudo-colored with the respective observed fluorescent emission signal.

### Measurement of cellular morphology and biofilm structure

Cell lengths and dimensions of biofilm structures were measured using Fiji as developed by the National Institutes of Health [106]. Analysis of cell length for planktonic and biofilm cell populations in Figure 3E included 2,000 cells each from three separate images collected from three independent replicates under identical conditions. Mid-exponential phase (OD _600 nm_ = 0.4 to 0.5) replicate planktonic cultures of strain H1206(pJAM1020) were imaged, diluted (to OD _600 nm_ = 0.1) in fresh medium (Hv-YPC +0.5% glucose), and 500 μl aliquots were transferred to replicate chamber-slide wells. Measurements were generated using the analyze particles function of Fiji [106].

### Preparation for macroscopic time-lapse studies and induction of social motility

#### Disruption and reformation

SL-biofilms shown in Figure 4, Additional file 3: Movie 2 and Additional file 4: Movie 3 were established by transferring the liquid culture (OD _600 nm_ = 1.0) into Petri dishes and incubating under static conditions for 7 days. These mature SL-biofilms were then mechanically homogenized through repetitive pipetting with a serological pipette to disrupt the layer of attached biofilm and large loosely attached aggregates. This was followed by transfer into a conical tube and gentle vortexing. The homogenized SL-biofilm culture was then transferred back into the original culture dish and left to incubate while photographs were collected.

#### Physical agitation

The SL-biofilm in Additional file 5: Movie 4 had formed after a liquid culture of *H. volcanii* H53 cells that had been freshly diluted in Hv-YPC medium (to OD _600 nm_ = 0.25) was transferred to Petri dishes and left to incubate for 5 days. Social motility as accessed by rapid formation of cellular extensions was induced in these biofilms by tapping the lid of the Petri dish, or by gently lifting and re-lowering the Petri dish approximately 1 cm. Such agitation did not disturb the overall biofilm; however, it caused smaller defects in loose biofilm structures, which were followed by prolonged social motility.

#### Control treatments

Established 7-day SL-biofilms were heat-treated within the culture dish at 75°C for 1 h or left untreated, but prepared for time-lapse observation identically (i.e., moved the same distance and partially disrupted during handling). Samples removed from heat-treated and untreated biofilms, stained with propidium iodide and prepared on microscope slides, verified that heat treatment left cells dead but intact, appearing like spheroplasts. Cells within SL-biofilms were also fixed by adding 37% formaldehyde (Fisher Chemical, Waltham, MA, F79-500) to a final concentration of 4% and incubating for 1 h prior to imaging as an additional control for cellular vitality.

### Biofilm recombination assay

Recombination within *H. volcanii* SL-biofilms was quantified using an approach adapted from a previously described method whereby two double auxotrophic strains are mixed and passed into nitrocellulose filters [56–58]. The strain H53, a uracil and tryptophan auxotroph, and the strain H98, a uracil and thymidine auxotroph (see Additional file 1: Table S2), where grown to late exponential phase (OD _600 nm_ 0.60 to 0.80) in Hv-YPC + thy, diluted (to OD _600 nm_ = 0.50), mixed together in equal parts and transferred into chamber slides or on top of filters for colony biofilm development.

SL-biofilms and colony biofilms were incubated and allowed to grow for 7 days, during which a biofilm developed (see Additional file 1: Figure S2). Static biofilms were then harvested by mechanical disruption through repetitive pipetting to detach the biofilm, followed by vortexing within a conical tube. Colony biofilms were placed into 1.5-ml tubes with 1 ml of Hv-starve medium with sterile forceps, vortexed and then shaken at 180 rpm for 1 h at 42°C to release cells for plating. An identical mixture of H53 and H98 liquid culture was also left shaking at 180 rpm for 7 days as a negative control. Samples for each condition were washed three times with Hv-starve medium, resuspended in Hv-starve and plated on Hv-Ca with uracil as selective screen for H53 and H98 prototrophic revertants and Hv-YPC + thy for total viable counts. Pure cultures of H53 and H98 were included for each condition as a negative control for mating. Plating on Hv-Ca medium without uracil was also included in the experiment to control for spontaneous reversion to uracil prototrophy (although this was not likely as the *pyrE2* gene is not mutated in H53/H98, but is completely deleted) or the presence of any ura + strain; no colonies were observed for any of these conditions. The transfer frequency was calculated by dividing the average number of colony-forming units (CFU) per ml on selective medium (Hv-Ca with uracil) for each replicate set by the corresponding averaged total CFU count (CFU/ml on Hv-YPC + thy medium).

## Additional files

Additional file 1:
**The file contains Figures S1 to S3 and Tables S1 to S3.** **Figure S1.** Colonies formed by *Haloferax volcanii* H1206(pSC409GFP). **Figure S2.** The basal layer of a 7-day H53/H98 mixed SL-biofilm stained with SYTO 9 at the time cells were plated for the recombination experiment shown in Figure 5. **Figure S3.** Seven-day *H. volcanii* H1206(pJAM1020) SL-biofilm stained with Congo red. **Table S1.** Cellular/matrix stains and fluorescent proteins used for biofilm visualization. **Table S2.** Strains and plasmids. **Table S3.** Oligonucleotide primers. Additional file 2: Movie 1. Time-lapse microscopy of long rods/adherent cellular structures within a developing 12-h *Haloferax volcanii* H1206(pJAM1020) SL-biofilm under blue excitation. Images were collected at 2-s intervals and replayed at 4 frames per second (8× real time). Additional file 3: Movie 2. Macroscopic time-lapse microscopy of a 7-day *Haloferax volcanii* H53 SL-biofilm following disruption and reformation. Fifteen hours of images are shown, collected after images shown in Figure 4, at 1-min intervals and replayed at 14 frames per second (840× real time). Additional file 4: Movie 3. Motility in wave-like patterns observed during macroscopic time-lapse photography of a 7-day *Haloferax volcanii* H53 SL-biofilm following disruption and reformation. Thirty minutes of images are shown (a subset of the data series in Movie 2) at 1-min intervals and replayed at 3 frames per second (180× real time). Additional file 5: Movie 4. Macroscopic time-lapse microscopy of a 5-day *Haloferax volcanii* H53 SL-biofilm. Ninety minutes of images are shown immediately following physical agitation without complete disruption of the biofilm at 10-s intervals and replayed at 30 frames per second (300× real time). Additional file 6: Movie 5. Macroscopic time-lapse microscopy of heat-treated (A) and untreated (B) 7-day *Haloferax volcanii* H53 SL-biofilms. Three hours of images are shown, collected at 30-s intervals and replayed at 14 frames per second (420× real time). Additional file 7: Movie 6. Macroscopic time-lapse microscopy of 7-day *Haloferax volcanii* H53 SL-biofilms treated with 4% formaldehyde (A) or left untreated (B). Eighty minutes of images are shown, collected at 30-s intervals and replayed at 14 frames per second (420× real time).

## Abbreviations

CLSM: confocal laser scanning microscopy
ConA: concanavalin A
CMO: CellMask Orange
CR: Congo red
CRF: Congo red fluorescence
DAPI: 4′,6-diamidino-2-phenylindole
DDAO: 7-hydroxy-9H-1-3-dichloro-9,9-dimenthylacridin-2-one
ECM: extracellular matrix
eDNA: extracellular DNA
EPS: extracellular polymeric substance
GFP: green fluorescent protein
HGT: horizontal gene transfer
PEG: poly(ethylene) glycol
SL-biofilm: static liquid biofilm
ThT: thioflavin T
