# Supplementary material for: Biofilms formed by the archaeon Haloferax volcanii exhibit cellular differentiation and social motility, and facilitate horizontal gene transfer
Source: BMC Biol. 2014 Aug 14;12:65. doi: 10.1186/s12915-014-0065-5 (PMC4180959; doi:10.1186/s12915-014-0065-5)
Supplement: Additional file 1: — The file contains Figures S1 to S3 and Tables S1 to S3. Figure S1. Colonies formed by Haloferax volcanii H1206(pSC409GFP). Figure S2. The basal layer of a 7-day H53/H98 mixed SL-biofilm stained with SYTO 9 at the time cells were plated for the recombination experiment shown in Figure 5. Figure S3. Seven-day H. volcanii H1206(pJAM1020) SL-biofilm stained with Congo red. Table S1. Cellular/matrix stains and fluorescent proteins used for biofilm visualization. Table S2. Strains and plasmids. Table S3. Oligonucleotide primers. [file 12915_2014_65_MOESM1_ESM.pdf]

Additional file 1: Supplemental figures and tables

Figures S1-S3

Tables S1-S3

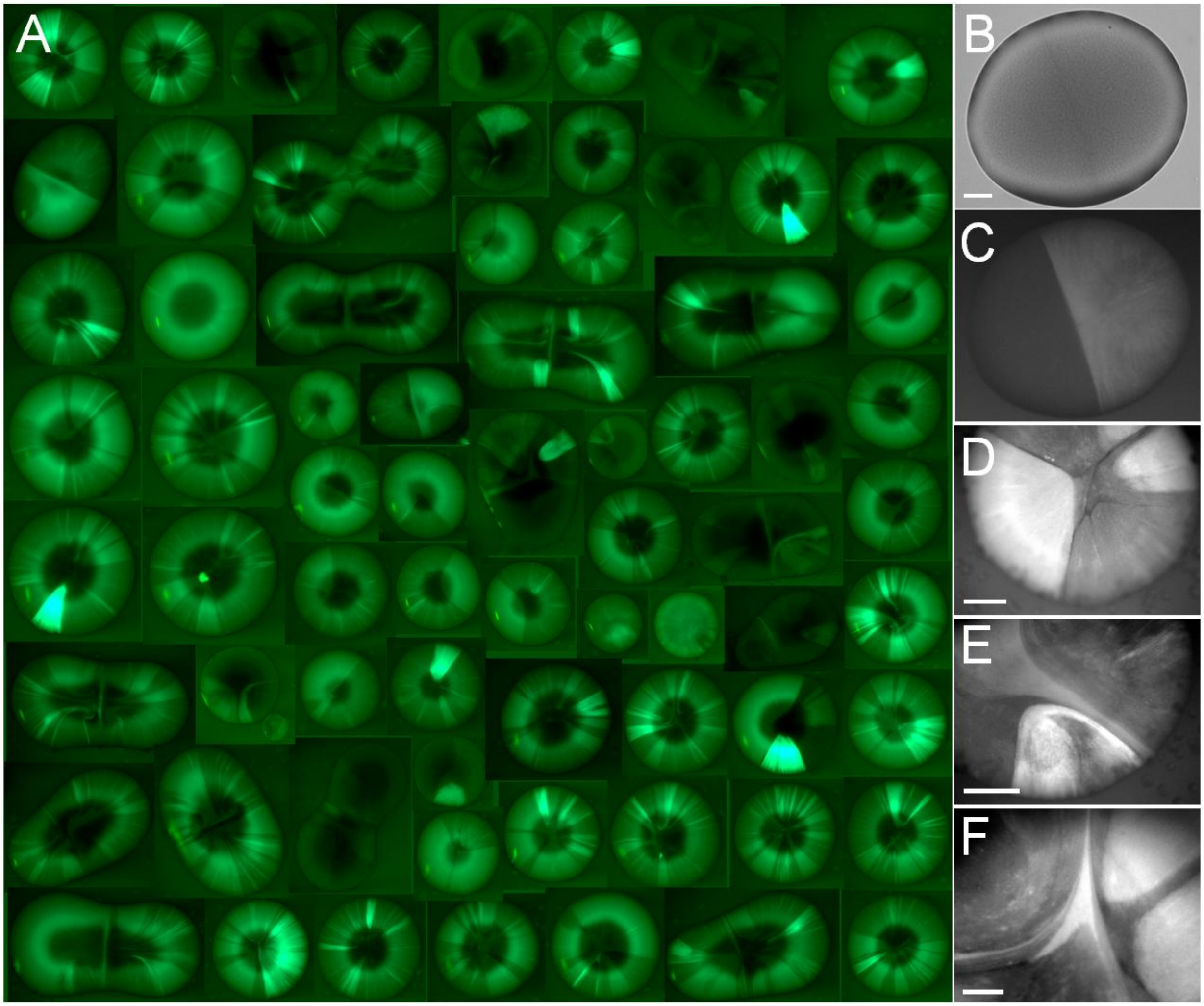

Figure S1 Colonies formed by *Hfx. volcanii* H1206(pSC409GFP). Patterns shown were present after repeated subculture. (**A**) Randomly selected population of seventy-eight colonies on selective medium (Hv-Ca without uracil) imaged with a fluorescence dissecting microscope. (**B-F**) Colonies like those in (**A**) imaged at higher magnifications under transmitted light (**B**) and with blue excitation (**C-F**). Scale bars for (**B-E**) equal 100 µm and 25 µm for (**F**).

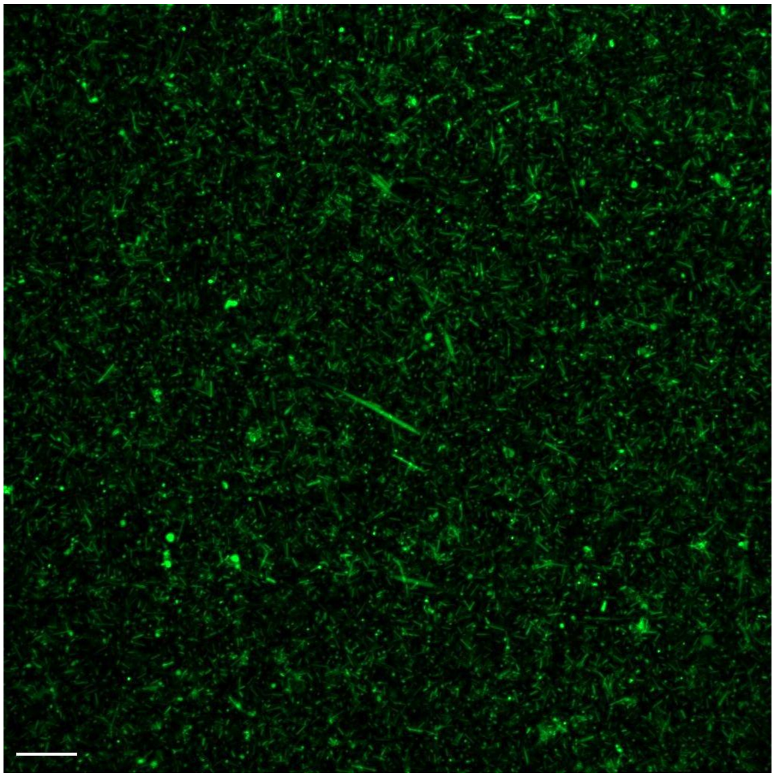

Figure S2 Projection for a Z-stack of the first 10 µm (or basal-layer) of a replicate 7-day *Hfx. volcanii* H53/H98 mixed SL-biofilm grown in a chamber slide and stained with SYTO 9 (imaged at the time cells were plated for recombination experiment shown in Figure 5). Scale bar equals 20 µm.

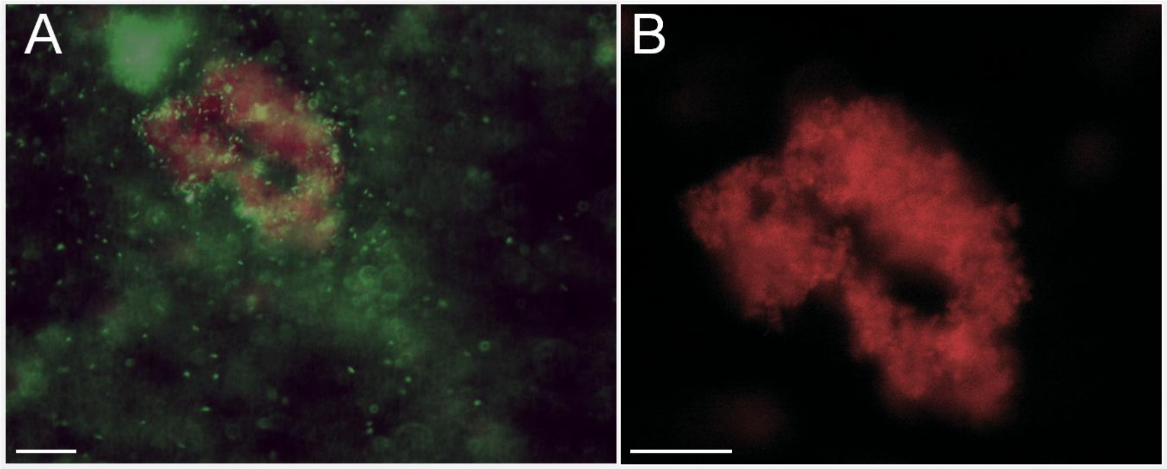

Figure S3 7-day *Hfx. volcanii* H1206(pJAM1020) SL-biofilm stained with Congo red. (**A**) Overlay of images taken under blue excitation for GFP signal and green excitation for Congo red fluorescence. (**B**) Higher magnification view from (**A**) of mature cluster displaying Congo red fluorescence. Scale bars equal 20 µm.

Table S1 Cellular/matrix stains and fluorescent proteins used for biofilm visualization.

Stain/fluorescent protein	Putative target/properties	Final concentration of staining solution	Excitation/ emission (nm)	Method reference/source
**Live cells**				
FM 1-43	Cell membrane, lipophilic	1 µg/ml	595/615	Molecular Probes, F10317
CellMask Orange (CMO)	Cell membrane, amphipathic	2 µg/ml	554/567	Molecular Probes, C10045
red-shifted GFP	constitutive endogenous signal		490/509	[1]; see Table 1
**Dead Cells**				
Propidium Iodide (PI)	Cellular DNA	30 µM	535/617	[2]; Molecular Probes, D3566
**Matrix components**				
DAPI	eDNA	5 µg/ml	350/470	[3, 4]; Molecular Probes, D1306
Congo red (CR)	amyloid fibrils	20 µM	497/614[a]	[5, 6]; Sigma, 573-58-0
Thioflavin T (ThT)	amyloid fibrils	1.5 µM	450/482[a]	[7]; Acros Organics, 211760050
Concanavalin A-Texas Red (ConA)	glycoconjugates	20 µg/ml	595/615	[3]; Molecular Probes, C825
**SYTO 9**	cellular DNA and eDNA	10 µM	485/498	Molecular Probes, S34854

[a] When bound to amyloid fibrils

Table S2 Strains and Plasmids.

Strain or Plasmid	Relevant properties	Reference or source
**Plasmids**		
pTA409	Shuttle vector based on pBluescript II, with *pyrE2* and *hdrB* markers and *ori-pHV1/4* replication origin	Hölzle et al., 2008
pJAM1020	Amp^R Nov^R; pHV2 replication origin; RSGFP expressed in *Hfx. volcanii;* rRNA P2 promoter and T7 terminator	Reuter, et al., 2004
pSC409GFP	pTA409 with 933 bp KpnI/EcoRI In-Fusion fragment containing RSGFP expression construct from pJAM1020	This study
**Strains**		
E. coli		
HST08	Stellar competent cloning strain	Clontech, 636763
K12	*E. coli dam dcm* cloning strain	New England Biolabs, C2925I
Hfx. volcanii		
DS2	Wild-type	Mullakhanbhai and Larsen, 1975; Hartman et al., 2010
H26	Δ*pyrE2*	Allers et al., 2004
H53	Δ*pyrE2* Δ*trpA*	Allers et al., 2004
H98	Δ*pyrE2* Δ*hdrB*	Allers et al., 2004
H1206	Δ*pyrE2* Δ*mrr*	Allers et al., 2010
H1206(pJAM1020)	Δ*pyrE2* Δ*mrr*, with pJAM1020; constitutive RSGFP expression; Nov^R	This study
H1206(pSC409GFP)	Δ*pyrE2* Δ*mrr*, with pSC409GFP; constitutive RSGFP expression	This study

Table S3 Oligionucleotide primers.

Primer	Sequence (5' -3')[a]	Properties
SC_P2promoter_F	TATAGGGCGAATTGGGTACCCA ACCCCCGATCCAAGCTTCTAGAG	Forward primer for amplification of RSGFP with P2 promoter and T7 terminator
SC_T7terminator_R	CGGGCTGCAGGAATTCTTCA GCAAAAAACCCCTCAAGAC	Forward primer for amplification of RSGFP with P2 promoter and T7 terminator

[a] In-Fusion adaptor sites are underlined.

Supplemental references

1. Reuter CJ, Maupin-Furlow JA: **Analysis of proteasome-dependent proteolysis in *Haloferax volcanii* cells, using short-lived green fluorescent proteins**. *Applied and environmental microbiology* 2004, **70**(12):7530-7538.
2. Leuko S, Legat A, Fendrihan S, Stan-Lotter H: **Evaluation of the LIVE/DEAD BacLight kit for detection of extremophilic archaea and visualization of microorganisms in environmental hypersaline samples**. *Applied and environmental microbiology* 2004, **70**(11):6884-6886.
3. Fröls S, Dyall-Smith M, Pfeifer F: **Biofilm formation by haloarchaea**. *Environmental microbiology* 2012, **14**(12):3159-3174.
4. Jurcisek JA, Bakaletz LO: **Biofilms formed by nontypeable Haemophilus influenzae in vivo contain both double-stranded DNA and type IV pilin protein**. *J Bacteriol* 2007, **189**(10):3868-3875.
5. Cegelski L, Pinkner JS, Hammer ND, Cusumano CK, Hung CS, Chorell E, Aberg V, Walker JN, Seed PC, Almqvist F *et al*: **Small-molecule inhibitors target *Escherichia coli* amyloid biogenesis and biofilm formation**. *Nature chemical biology* 2009, **5**(12):913-919.
6. Puchtler H, Waldrop FS, Meloan SN: **A review of light, polarization and fluorescence microscopic methods for amyloid**. *Appl Pathol* 1985, **3**:5-17.
7. Larsen P, Nielsen JL, Dueholm MS, Wetzel R, Otzen D, Nielsen PH: **Amyloid adhesins are abundant in natural biofilms**. *Environmental microbiology* 2007, **9**(12):3077-3090.
